# Meaningfulness protects from and crisis of meaning exacerbates general mental distress longitudinally

**DOI:** 10.1186/s12888-022-03921-3

**Published:** 2022-04-21

**Authors:** Tatjana Schnell, Henning Krampe

**Affiliations:** 1 Psychology of Religion and Existential Psychology, Social Sciences, MF Specialized University, Gydas vei 4, 0363 Oslo, Norway; 2 Institute of Psychology, Existential Psychology Lab, University of Innsbruck, Innrain 52, 6020 Innsbruck, Austria; 3 Department of Anesthesiology and Operative Intensive Care Medicine (CCM, CVK), Charité - Universitätsmedizin, corporate member of Freie Universität Berlin, Humboldt-Universität zu Berlin, and Berlin Institute of Health, Berlin, Germany

**Keywords:** COVID-19,, General mental distress, Meaning in life, Crisis of meaning, prospective study, Longitudinal, Within-subject mediation

## Abstract

**Background:**

Reactions to the COVID-19 pandemic are diverse, and both mental distress and existential crises can arise. The identification of protective and exacerbating factors and their progress over time is therefore highly relevant. The current study examined longitudinal protective effects of meaningfulness and exacerbating effects of crisis of meaning on general mental distress.

**Methods:**

*N* = 431 participants from Germany and Austria (mean age: 42 years) completed an online survey in both April/May (T1) and July/August 2020 (T2). After determining temporal stability or changes in meaningfulness, crisis of meaning, and general mental distress (PHQ-4), we examined whether (i) meaningfulness and (ii) crisis of meaning, measured at T1, incrementally predicted PHQ-4 at T2, beyond baseline levels of PHQ-4. We further tested (iii) a within-subject mediation of temporal changes in PHQ-4 by changes in crisis of meaning.

**Results:**

Meaningfulness prospectively predicted lower PHQ-4, and crisis of meaning predicted higher PHQ-4. From the first wave of the pandemic until a slowdown three months later, meaningfulness was stable, and crisis of meaning and PHQ-4 decreased. Changes in crisis of meaning mediated the changes in PHQ-4.

**Conclusions:**

Meaningfulness appears to have a protective, and crisis of meaning an exacerbating effect on psychological distress, as shown here for the time of the first pandemic wave until three months later. Attention to existential experiences of meaningfulness and loss of meaning thus proves relevant to the clinical and public health context. Measures that support meaningfulness will help coping with crises of meaning, which in turn supports overcoming general mental distress.

## Background

In March 2020, the World Health Organization (WHO) declared the Coronavirus disease 2019 (COVID-19) to be a pandemic. The early phase of the pandemic was accompanied by increased levels of mental distress in the general population in Germany and Austria as in many other countries [1–7]. Several relevant resources were identified that appeared to serve as protective factors. Older age [8, 9] and male gender [10, 11] were associated with higher mental stability in terms of demographic characteristics. Supportive psychological characteristics were trust in the healthcare system [12, 13], psychological flexibility and acceptance of difficult experiences [14], self-esteem [15], resilience [16], and self-control [2, 17].

Beyond threatening mental health, large-scale crises also have the potential to shatter worldviews, jeopardize existential security, and trigger crises of meaning [18–20]. Several researchers have addressed existential experiences during the pandemic. Besides an intensified confrontation with one's mortality [21, 22], experiences of meaning – or its loss – are of crucial importance here. In times of crisis, meaningfulness is a resource that fundamentally determines whether a person sees their life as worth living and is therefore willing and motivated to actively overcome challenges and take responsibility for their health [20]. Various studies have shown that meaning in life was a protective factor during the pandemic [2, 23–26]. At the same time, it emerged that for quite a few people, the large-scale crisis was accompanied by a shaking of their worldview [27]. High levels of acute stress caused by the pandemic were linked to crises of meaning, which in turn predicted high levels of general mental distress [2]. The latter study also found that meaningfulness and crisis of meaning covaried with the pandemic-related restrictions in Germany and Austria. Meaningfulness was high and crisis of meaning low during the first lockdown, which met with very high approval from the population [28]. Meaningfulness was substantially lower and crisis of meaning higher for the second survey period covering the weeks immediately following the lockdown, a time characterized by insecurity, contradictions in the communication of public health measures, and regional differences regarding the measures in force.

This suggests that public health measures—or the way they are communicated and implemented—may have far-reaching consequences in the lives of individuals [29]. Furthermore, there is evidence that psychological distress occurred primarily at the beginning of the pandemic and decreased in the following months for the majority of respondents [30–32]. For some, however, stress levels either persisted or even increased [33, 34]. It is therefore a major research goal to identify not only resources that contribute to resilience, but also the risk factors that sustain or exacerbate mental distress. Since these are temporal processes, longitudinal data are of particular importance.

As far as the role of meaning in life is concerned, protective effects have been confirmed in longitudinal studies among Chinese students [35, 36]. To our knowledge, studies in the general population and other countries are still pending, and implications of a lack of meaning have not been studied longitudinally at all. The present study thus aimed to examine the protective and exacerbating effects of meaning in life (meaningfulness and crisis of meaning) on general mental distress in a longitudinal design. Based on evidence that meaningfulness had not changed from the onset of the pandemic to three months later, but crisis of meaning and general mental distress had decreased [37], we first investigated whether meaningfulness prospectively predicted lower, and crisis of meaning higher general mental distress. To further gain insight into temporal change processes at the person level, we examined a within-subject mediation effect for crisis of meaning. Building on findings by Schnell & Krampe (2020) [2], we tested the following hypotheses: (i) Meaningfulness as measured during the first wave of the pandemic (T1) serves as a negative predictor of general mental distress as measured three months later (T2), beyond baseline levels of general mental distress (T1). (ii) Crisis of meaning as measured during the first wave of the pandemic (T1) serves as a positive predictor of general mental distress as measured three months later (T2), beyond baseline levels of general mental distress (T1). (iii) The slowdown of the pandemic was accompanied by changes in crisis of meaning, which in turn mediated the effect of time on general mental distress.

## Methods

### Procedure and participants

Online surveys were conducted during the first wave of the pandemic in April/May 2020 (T1) and in a period of relatively low incidence in July/August 2020 (T2). All T1 participants who had agreed to be contacted again were invited to participate in the follow-up study. Inclusion criteria at T1 were providing informed consent and a minimum age of 18 years; consent to repeat participation was not an inclusion criterion. Exclusion criteria were incomplete questionnaires and not affirming honest reporting. *N* = 1,568 participants completed the questionnaire at T1, *N* = 431 took part at T2. Evidence of biased attrition was found for education only: Participants at T2 were slightly more educated, odds ratio = 1.21 (standardized predictors). Among those who participated twice, thirty-four percent reported secondary or advanced education, 66% had a university degree. Sixty-six percent identified as women and 34% as men. The mean age was 42 (SD = 17; two missing values), ranging from 18 to 82 years. Fifty-three percent were resident in Germany, 41% in Austria, the remainder in Switzerland or Italy.

### Measures

Two dimensions of meaning in life, meaningfulness and crisis of meaning, were assessed at T1 and T2 using the respective 5-item scales of the Sources of Meaning and Meaning in Life Questionnaire (SoMe; [38, 39]) with a six-point Likert scale (0–5). Cronbach's alphas were 0.81 and 0.83 for meaningfulness, and 0.92 and 0.94 for crisis of meaning. Also at T1 and T2, general mental distress was measured by the Patient Health Questionnaire-4 (PHQ-4) [40], a brief four-item measure of core symptoms of depression and anxiety (four-point Likert scale, 0–3). Cronbach's alphas were 0.84 and 0.83, respectively. While the original version of the SoMe [38, 39] was developed in German language, we employed a validated German translation of the PHQ-4 [41, 42].

### Analysis

Descriptive statistics including Cronbach’s alphas, means, standard deviations, and paired-sample t-tests were used to describe the sample and temporal changes. Gender, age, and education were examined as potential confounders. To test the hypotheses that meaningfulness protects from, and crisis of meaning exacerbates general mental distress three months later, we performed hierarchical linear regression analyses controlling for covariates and PHQ-4 at T1. The analyses were conducted separately for meaningfulness and crisis of meaning, as both are substantially correlated, but represent relatively independent dimensions [39, 43]. This is reflected in the fact that a decrease in meaningfulness is not necessarily associated with an increased crisis of meaning [44].

Moving on to the third hypothesis, we estimated a within-subject mediation model using MEMORE (Mediation and Moderation Analysis for Repeated Measures Designs) [45], version 2.1, model 1). Here, the mediator was the difference between measurements of crisis of meaning at T1 and T2. The outcome was the difference between measures of general mental distress at T1 and T2. The model thus tested if changes in the mediator (crisis of meaning) were associated with changes in mental distress (PHQ-4) from an early to a later phase of the pandemic. The grand mean-centered mean of the mediator pair was used as a covariate. We set bootstrapping at 5,000 samples and estimated percentile 95% confidence intervals.

We had planned to include all participants who responded to our invitation, aiming to collect the largest sample size possible. According to several scholars (e.g. [46]), this strategy might be preferable to power analyses with a focus on traditional null hypothesis significance testing. Since this information is after all frequently requested, we report the result of a post-hoc power analysis for hierarchical linear regression analyses: With the given sample size of *N* = 431 and an α = 0.05, the power to detect a small (f^2^ = 0.02) increase in R^2^ was sufficient at 0.83 ([G*Power [47]; version 3.1.9.6]).

Power analysis for within-subject mediation designs is complex. According to Montoya (2021), within-subject designs typically require half the sample size of between-subject designs to detect indirect effects of the same size [48]. However, power is dependent on the correlations among repeated measurements, with a peak of power associated with a correlation of ρ_M_ = 0.75 among the mediators, but increasing power with increasing correlation of the outcome variables. Moreover, power seems to benefit from estimating the moderation parameter (the grand mean-centered mean of the mediator pair). Finally, Montoya concludes that in a within-subject design, between 100 and 200 participants suffice to achieve the statistical power of 0.80 for an indirect effect of 0.15 when using bootstrap confidence intervals, but to detect smaller effects, larger sample sizes are needed [48].

## Results

Table 1 shows descriptive statistics and paired-sample t-tests. Skewness and kurtosis values for all variables indicated near-normal data distribution ( <|2|, not shown; [49]).

**Table 1:** Descriptive statistics and paired sample t-tests

Variable	α_T1_	M/SD _T1_	α _T2_	M/SD _T2_	r_T1-T2_	t(430)	*d*_*HC*_^c^	95% CI for *d*_*HC*_* (LL, UL)*
Meaningfulness^a^	.81	3.01/1.16	.83	3.07/1.12	.76	-1.59	-.08	-.171, 0.18
Crisis of meaning^a^	.92	1.08/1.26	.94	0.96/1.20	.72	**2.80**	.14	.040, .230
General mental distress^b^	.84	3.29/2.78	.83	2.97/2.59	.61	**2.75**	.13	.037, .227

Note. *N* = 431

^a ^range = 0–5

^b ^PHQ-4 sum score, range = 0–12

^c ^Cohen’s d with Hedges’ correction

*T1* Time 1, *T2* Time 2. Bold = significant at *p* = .006 (two-sided)

Measures at T1 and T2 were highly correlated (*r* = 0.61—0.76). Paired sample t-tests showed significant but small decreases in crisis of meaning (*Cohen’s d with Hedges’ correction* = 0.14) and general mental distress (*Cohen’s d with Hedges’ correction* = 0.13). Meaningfulness did not change over the course of the three months.

Table 2 displays scale intercorrelations and examines age, gender, and education as potential confounders.

**Table 2:** Correlations between scales, age, gender, and education

	(1)	(2)	(3)	(4)	(5)	(6)
(1) Meaningfulness (T1)		.76**	-.65**	-.56**	-.43**	-.34**
(2) Meaningfulness (T2)			-.62**	-.72**	-.41**	-.47**
(3) Crisis of meaning (T1)				.72**	.64**	.48**
(4) Crisis of meaning (T2)					.51**	.63**
(5) General mental distress (T1)						.61**
(6) General mental distress (T2)						
Age	.03	.06	-.03	-.03	-.09	-.15**
Gender^a^	.18**	.22**	-.10*	-.15**	.01	.01
Education^b^	.13**	.16**	-.08	-.10*	-.08	-.11*

Note. *N* = 431. *T1* Time 1, *T2* Time 2

^a ^0 = male, 1 = female

^b ^0 = advanced or less, 1 = university degree

^*^ *p* < .05 (two-sided). ** *p* < .01 (two-sided)

Age and education covaried with the dependent variable, general mental distress, at T2. In the following hierarchical linear regressions, they were thus included as covariates.

### Examining longitudinal protective and exacerbating effects of meaningfulness and crisis of meaning on general mental distress

Table 3 shows two hierarchical regression analyses to longitudinally predict general mental distress. Models 1 and 2 are the same in both. For the first block, the results revealed a significant model (*p* = 0.002); age and education predicted approximately 3% of the variance in PHQ-4 at T2, with age the only significant predictor (β = -0.14, *p* = 0.004). The inclusion of PHQ-4 at T1 in the second block (β = 0.60, *p* < 0.001) led to a significant increase in the variance accounted for by the model (R^2^ change = 0.36, *p* < 0.001). Including meaningfulness in the third block (β = -0.10, *p* = 0.02) resulted in an additional increase in explained variance (R^2^ change = 0.008, *p* = 0.02). Including crisis of meaning in the third block of the second hierarchic regression analysis (β = 0.15, *p* = 0.002) also resulted in an additional increase in explained variance (R^2^ change = 0.014, *p* = 0.002).

**Table 3:** Two hierarchical regression analyses to longitudinally predict general mental distress (T2)

Predictors	*B*	*SE B*	*95% CI*	*β*	R^2^	ΔR^2^
Step 1					.029	.029
Age	-.02	.01	(-.04, -.01)	-.14**		
Education^a^	-.42	.27	(-.95, .10)	-.08		
Step 2					.388	.358
Age	-.02	.01	(-.03, -.00)	-.09*		
Education^a^	-.20	.21	(-.62, .21)	-.04		
General mental distress (T1)	.56	.04	(.49, .63)	.60***		
Step 3—**Meaningfulness**					.396	.008
Age	-.02	.01	(-.03, -.00)	-.14**		
Education^a^	-.15	.21	(-.56, .27)	-.03		
General mental distress (T1)	.52	.04	(.44, .60)	.56***		
Meaningfulness (T1)	-.23	.09	(-.41, -.04)	-.10*		
Step 3 – **Crisis of meaning**					.401	.014
Age	-.02	.01	(-.03, -.00)	-.10*		
Education^a^	-.17	.21	(-.58, .24)	-.03		
General mental distress (T1)	.47	.05	(.38, .56)	.50***		
Crisis of meaning (T1)	.31	.10	(.11, .51)	.15**		

Note. *N* = 431. *T1* Time 1, *T2* Time 2. 95% CI = 95% confidence interval for estimate B (lower limit, upper limit). ^a ^0 = advanced or less, 1 = university degree

^*^ *p* < .05. ** *p* < .01. *** *p* < .001

**Fig. 1:**
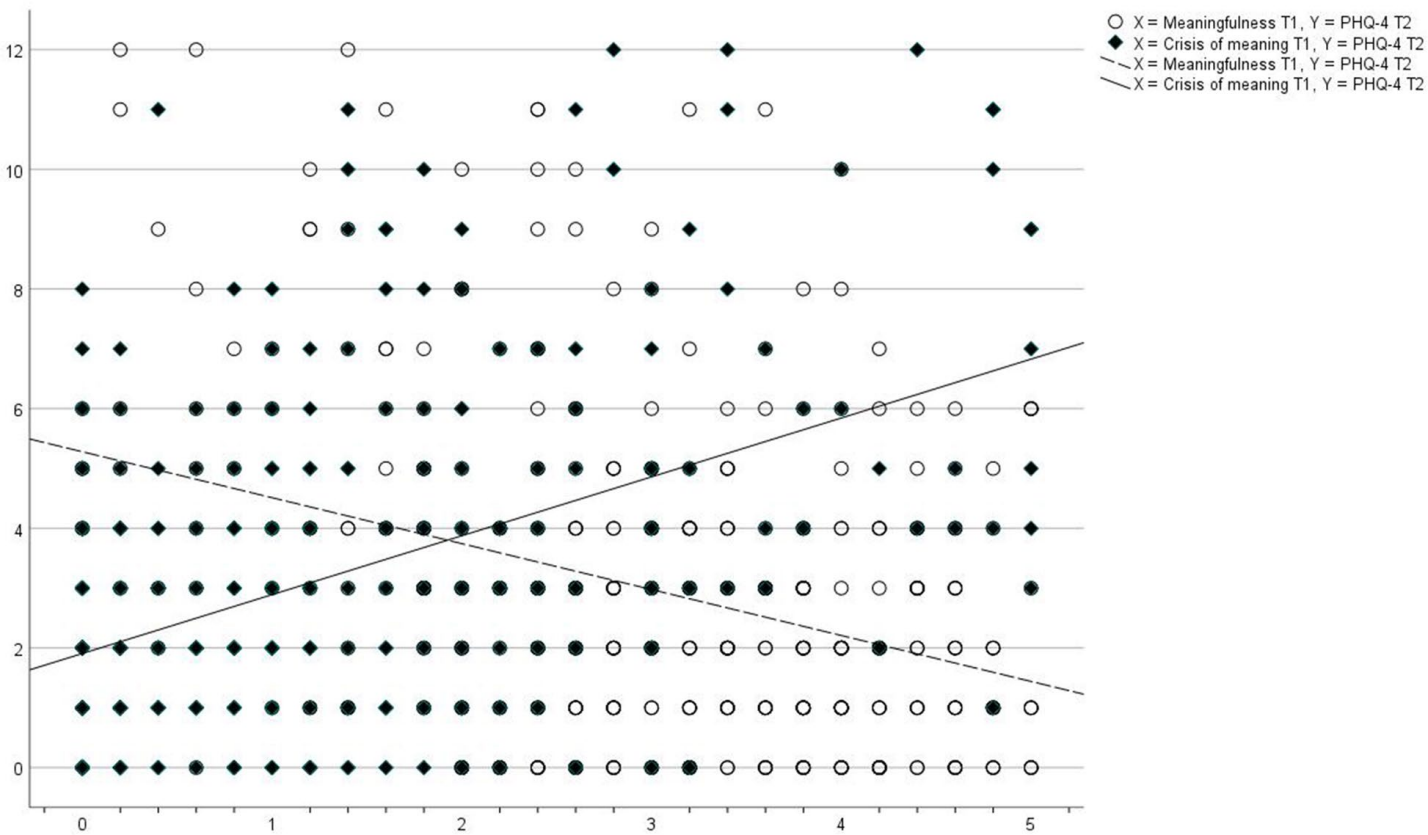
Overlay scatterplot with linear fit lines for the associations between (i) meaningfulness (T1) and PHQ-4 (T2), and (ii) crisis of meaning (T1) and PHQ-4 (T2). *Note.* X-axis = meaningfulness/crisis of meaning (T1). Y-axis: General mental distress (PHQ-4)(T2)

Figure 1 shows an overlay scatterplot with linear fit lines for the associations between meaningfulness (T1) and PHQ-4 (T2), and crisis of meaning (T1) and PHQ-4 (T2). Severe symptoms of depression and anxiety (values > 6 [40]) are mainly observed at low scores in meaningfulness and high scores in crisis of meaning. The cut-off score for moderate symptoms (> 4 [42]) is exceeded at values > 2.10 in crisis of meaning and values < 1.60 in meaningfulness. Low scores in meaningfulness are not associated with severe symptoms, but high scores in crisis of meaning are.

### Examining a within-subject mediation effect of changes in crisis of meaning on changes in general mental distress

**Fig. 2:**
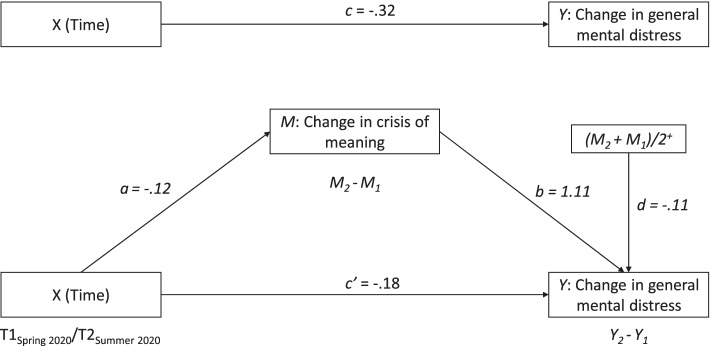
Within-subject mediation model for the effect of time on changes in general mental distress through changes in crisis of meaning. *Note.* The superscript ^+^ indicates grand mean-centered. T1 = Time 1, first wave of the pandemic in spring 2020. T2 = Time 2, three months later in summer 2020

Utilising MEMORE [45], we tested the indirect effect of time on general mental distress through changes in crisis of meaning. Figure 2 displays the within-subject mediation model and corresponding regression coefficients for each pathway.

The results showed that the total effect of time on PHQ-4 was significant (c = -0.316 [-0.542, -0.090], t(430) = -2.75, *p* = 0.006), meaning that general mental distress decreased in the period from the onset of the pandemic to a time of less infections and lowered restrictions, three months later. With time, also crisis of meaning decreased significantly (a = -0.12 [-0.211, -0.037], t(430) = -2.80, *p* = 0.005). The changes in crisis of meaning, in turn, were positively related to the changes in PHQ-4 scores (b = 1.11 [0.885, 1.332], t(428) = 9.76, *p* < 0.001). As hypothesized, there was a significant indirect effect of time on PHQ-4 through changes in crisis of meaning (ab = -0.14 [-0.254, -0.039], ps (partially standardized indirect effect) = -0.06, proportion of the total effect due to the indirect effect = 44%. This indicates that improvements with regard to crisis of meaning accounted for a substantial proportion of improvement in general mental distress.

## Discussion

The question of whether or not we perceive our lives as meaningful has profound implications for how we relate to ourselves and our environment [20]. The evaluation of life as meaningful determines whether we see life as worth living at all and are thus motivated to invest in constructive interaction with the environment—even if this should be challenging [50]. Apart from this activating and motivating function which has been replicated by several studies (e.g. [51, 52],), meaningfulness also has a protective function: It impacts how people cope with stress or pain [53, 54]. A crisis of meaning, on the other hand, is a state of severe existential insecurity. People who suffer from not seeing meaning in their lives do not have access to personal resources such as hope, self-efficacy, or resilience; instead, depression, anxiety, negative mood and pessimism prevail [43, 55–57]. Despite the overlap with clinical symptoms, crises of meaning cannot be explained by these alone. Thus, most respondents who suffer from depression also tend to report high scores in crisis of meaning—but the reverse is true to a much lesser extent [58]. A crisis of meaning further proved to be a significant predictor of suicidality when controlling for depression [58], which demonstrates that the prevalence of crises of meaning in the population should be seriously monitored.

The present study looked at both the protective effect of meaningfulness and the risk to mental health associated with crisis of meaning. Its primary aim was to understand effects over time. The results confirmed our hypothesis that people who reported higher levels of meaningfulness at the beginning of the pandemic suffered from less general mental distress three months later. The bivariate prospective correlation exhibited a negative effect of medium size; when additionally controlling for age, education and general mental distress at the first measurement point, the incremental predictive value of meaningfulness was still significant. Our study is thus in line with recent research that showed a risk-protective effect of meaning in life [35, 36]. Testing the second hypothesis confirmed a reverse effect: Individuals who reported higher crisis of meaning scores at the beginning of the pandemic suffered more general mental distress three months later—even when controlling for demographic covariates and baseline PHQ-4 score. Here, a high level of crisis of meaning predicted more severe symptoms of depression and anxiety than a low level of meaningfulness. This confirms earlier findings [44] that the mere absence of meaning does not necessarily imply suffering and stress. Above all, it is the suffering from a meaningless life that leads to further symptoms.

The third hypothesis tested in the present study focused on the changes that occurred during the first months of the pandemic. Using paired-sample tests, meaningfulness was found to be stable across the two measurement points—replicating previous evidence of its stability [39, 59]. A sense of meaning in life is thus not easily shaken, apparently not even by the occurrence of a pandemic—at least as far as the first months of the emergence of the coronavirus disease are concerned. The extent of crisis of meaning measured during the first wave of infection, on the other hand, declined slightly in the following three months. This was also true for general mental distress. The third hypothesis therefore referred to these two characteristics. Based on the assumption that crises of meaning can lead to psychological distress, we tested whether their decrease would also predict the decrease in symptoms of distress. The corresponding indirect effect proved to be significant. The proportion of the total effect attributable to the indirect effect was substantial at 44%. This suggests that existential concerns should not be disregarded in clinical and public health contexts. It seems feasible to prevent mental distress and support mental health by addressing a crisis of meaning, e.g. through counselling or therapy.

The outcomes of the present study are in line with the general scientific evidence. Considering the overall responses to the pandemic, the majority seems to be resilient, whereas some experience it as a critical interruption of the continuity of their life [60]. According to our and other published [30–32] data, this might be a short-term crisis, as both scores in crisis of meaning and general mental distress decreased after the onset of the pandemic. Nevertheless, elevated scores of crisis of meaning during the first wave of the pandemic predicted higher general mental distress three months later. This suggests that a significant minority questions social or personal priorities [27] and enters into a crisis of meaning [2]. Although such crises are typically accompanied by psychological suffering [55–57] and even suicidality [58], they also have a considerable constructive potential: A more authentic approach to life, based on a more realistic—and thus more stable – worldview, seems to come into effect when crises are genuinely confronted [61, 62].

### Strengths and limitations

This study's strengths include a longitudinal design with a substantial sample size and the employment of validated measures to assess two dimensions of meaning in life and general mental distress. Its major limitation is the fact that the sample is not representative. We did not use random sampling, and women and more educated participants were over-represented. This was considered in the analyses by including education as a covariate, but not gender, as it was not related to the outcome variable, general mental distress. Second, there was a slightly greater risk of dropout amongst individuals with lower education. However, this effect was small, and since the study focused on within-subject changes, we assume that attrition did not lead to any relevant bias. Finally, we cannot draw robust conclusions about causality. Studies with two-wave designs reduce the chance of bias due to common methods [63], but studies with more than two waves will allow for higher fidelity of conclusions about the proposed indirect effect [64].

## Conclusion

Summarising the results indicates that existential questions should be taken seriously and targeted in times of large-scale crises—both in counselling and therapy and concerning public health measures. Public health guidelines can have a tangible impact on the four pillars of meaningfulness—significance, purpose, coherence, and belonging [20] through the design of the measures taken, their communication, and implementation [29]. Our data show that enabling citizens to maintain meaning in their lives even under challenging conditions is an effective preventive measure against the emergence of mental health problems. Elevated levels of a crisis of meaning, on the other hand, prospectively increased the likelihood of experiencing symptoms of depression and anxiety, while coping with them also proved beneficial for the progression of general mental distress.

## Data Availability

The dataset generated and analysed during the current study is not publicly available because participants did not agree for their data to be shared publicly, but it is available from the corresponding author on reasonable request.
